# Giving Is Nicer than Taking: Preschoolers Reciprocate Based on the Social Intentions of the Distributor

**DOI:** 10.1371/journal.pone.0147539

**Published:** 2016-01-25

**Authors:** Martina Vogelsang, Michael Tomasello

**Affiliations:** 1 Developmental Psychology Group, Department of Psychology, University of Kassel, Kassel, Germany; 2 Department of Developmental and Comparative Psychology, Max-Planck-Institute for Evolutionary Anthropology, Leipzig, Germany; University of Bologna, ITALY

## Abstract

Recent research has found that even preschoolers give more resources to others who have previously given resources to them, but the psychological bases of this reciprocity are unknown. In our study, a puppet distributed resources between herself and a child by taking some from a pile in front of the child or else by giving some from a pile in front of herself. Although the resulting distributions were identical, three- and five-year-olds reciprocated less generously when the puppet had taken rather than given resources. This suggests that children’s judgments about resource distribution are more about the social intentions of the distributor and the social framing of the distributional act than about the amount of resources obtained. In order to rule out that the differences in the children’s reciprocal behavior were merely due to experiencing gains and losses, we conducted a follow-up study. Here, three- and-five year olds won or lost resources in a lottery draw and could then freely give or take resources to/from a puppet, respectively. In this study, they did not respond differently after winning vs. losing resources.

## Introduction

When preschool children are given resources and are told to share them with another child as they like (e.g., in a dictator game), they tend to be mostly selfish, and this tendency is stronger the younger the children are [1] (see [2], for a review). However, recent research has found that young children are more generous if someone has just given something to them first. This tendency toward reciprocity is apparent in children as young as three years of age ([3; 4; 5; 6; 7;]; see [8] and [9] for research on reciprocity with older children).

One fundamental question about how people distribute resources in general concerns the relative role of the amounts of resources that are shared versus the social intentions and relationships that acts of distribution manifest. For example, adults in social psychology experiments (and also in real life) are content with a small number of resources, but they are unhappy with that same number of resources if others are getting more (see [10], for a review of this and similar research on social comparison processes). On the other hand, people are willing to accept fewer resources than others if they see that this outcome was the result of a fair procedure in which their needs and concerns were valued equally with everyone else’s (see [11], for a review of this and similar research on so-called procedural justice; see [12], for a study of procedural justice with children). Phenomena such as social comparison and procedural justice have led some social theorists to posit that acts of resource distribution are less about the instrumental value of resources than about the social dimensions of the distributive acts. For example, [13] gives an account in terms of the social recognition and respect for others that acts of distribution make manifest.

A finding with similar implications was reported by [14] in several experiments on reciprocity in adults. In the simplest contrast of conditions, the authors asked a confederate to distribute the resources at 50% for each player, but he did so either (a) by giving the subject $50 of $100 available in a computerized game, or else (ii) by taking $50 from the subjects $100. The clear finding was that subjects reciprocated less in the condition in which resources were taken from them than in the condition in which resources were given to them, even though the numerical distribution was identical in both conditions. The other experiments of [14] confirm this finding also in cases where the distributions were unequal (30 vs. 70%) and when the game was played over multiple rounds. This study helps to clarify some of the psychological motivations underlying reciprocity in resource distribution by documenting—once again but differently—that it is not primarily about the instrumental value of the resources per se. In this case, it seems to be about the social intentions of the original distributor as she goes about distributing.

One explanation of this result that avoids the notion of intentions (as well as those of social comparison studies, though not obviously of those of procedural justice studies) is that individuals are sensitive to so-called framing effects in which a resource distribution is seen as either a personal loss or gain, with distributions framed as a personal loss viewed negatively based on individual attitudes of loss aversion and/or an endowment effect [15; 16; 17]. The alternative is to recognize framing effects that are not based on personal loss or gain, but on whether the distributional act is framed as an act underlain by bad social intentions (e.g., taking something from another person) or good social intentions (e.g., giving something to another person).

In the current study, we adapted the method of [14] to test preschool children’s reciprocal behavior after being given resources versus after having resources taken from them. If children this young are simply operating with some kind of rote algorithm of equality in distribution—or some kind of "like for like" in reciprocity (e.g., she gave me three so I should give her three)—then it should not matter how a distribution is effected. But if they already see the act of distribution as a social act manifesting how the distributor views and/or evaluates them—as a kind of social framing effect—then it might be expected that they, like adults, would respond differently to identical distributions depending on whether they were effected by an act of giving or by an act of taking. We also ran a follow up study (Study 2) which was designed to rule out that children only reciprocated differently when being given resources vs. having resources taken from them is a result of merely experiencing a personal loss or gain, here operationalized through a lottery draw.

## Study 1

### Methods

#### Ethics statement

The presented study was non-invasive and strictly adhered to the legal requirements of the country in which it was conducted. The study and its follow-up were approved by the Max Planck Institute for Evolutionary Anthropology Ethics Committee. The full procedure of the study was covered by the committee’s approval. Informed written consent was obtained from all the parents of the children who participated in this study as well as in the follow-up (Study 2).

#### Participants

Children whose parents had previously given written consent were recruited from and tested in various kindergartens in Leipzig, Germany. Seventy-two children of three years of age and 72 children of five years of age took part in this study (36 girls, 36 boys in each age group). The three-year-olds age ranged from 36 to 41 months with a mean age of 38.75 months (SD 1.59 months). The five-year-olds age ranged from 60 to 65 months with a mean age of 62.43 months (SD 1.83 months). The children were from broadly middle-class backgrounds. Nineteen more children were tested but had to be dropped from the sample for various reasons: Five three-year-olds and two five-year-olds failed to follow instructions, two three-year-olds dropped out because of a mistake by the experimenters, and ten three-year-olds would not participate in the game.

#### Study setup and design

Study materials consisted of a hand puppet (45 cm tall), a blue and a white placemat, two small plastic dishes, two opaque plastic boxes, a memory game, and gummy bear candies. Each child was introduced to the experimenter and a puppet named Lola (played by the second experimenter) in her classroom and then went to the study room with them. Both experimenters were female. In the study room, the child, Lola and the experimenter played a memory-like game for a warm up. After that, the experimenter announced that they would now do something different and asked the child to sit down at the table in front of the blue felt placemat and Lola to sit down in front of the white one. Lola and the child were thus facing each other at the table. The experimenter then drew the attention of Lola and the child to the plastic boxes, telling them that both have their own box and something would go in there. She then said that they would now start and the reciprocal game began. After four complete rounds, the experimenter asked Lola and the child to show them how many gummy bears they had and exchanged those for new ones.

There were six experimental conditions: Three in which the children experienced Lola giving them candies (either 3 of 10; 5 of 10; or 7 of 10), and three in which the children experienced Lola taking candies from them (either 3 of 10; 5 of 10; or 7 of 10). Each child took part in only one condition, which results in 12 children per condition and age group. The child was always given the opportunity to reciprocate in the same manner as Lola, that is, for any particular child if Lola gave, then the child reciprocated by giving (and the same for taking).

In the three giving conditions, *give 3*, *give 5*, and *give 7*, the puppet Lola always began first. The experimenter took her plastic dish, filled it with ten gummy bears, and returned it to Lola, saying that she gets these gummy bears (which were said to be “hers”), and she could now give some to the child. The experimenter left the room and watched the scene over a Mini-DV-recorder that was located outside the room. At this point, Lola either gave three, five or seven gummy bears to the child by placing them into the child’s plastic dish. The experimenter returned to the room and asked Lola and the child how many gummy bears they now had and then to put them into their plastic boxes. Then the child received ten new gummy bears—in the same manner in which the puppet had received them before—and was told that she could now give some to Lola. Again, the experimenter left the room and returned after the child was done.

In the three taking conditions, *take 3*, *take 5* and *take 7*, the child first received ten gummy bears which were said to be hers. The experimenter then turned towards Lola and told her that she could take some from the child. After the experimenter had left the room, Lola then either took three, five or seven gummy bears from the child. When the experimenter returned she asked Lola and the child how many gummy bears they now had and that they could put them in their plastic boxes. Then Lola received ten new gummy bears and the child was told that she could take some from Lola. Again, the experimenter left the room and returned after the child was done.

In total, four complete rounds for the five-year-olds, and five for the three-year-olds were played. For each child, Lola always gave or took the same amount of gummy bears (that is, three, five or seven). In both the giving and taking conditions, the three-year-olds played an additional round with the puppet because a pilot study had shown that they needed some more warm-up with the distributing situation. We later checked whether their behavior in round 1 differed significantly from their behavior in the following four rounds, but it did not, *t*(71) = -1.78, *p* = 0.079, *d* = -0.219, two-tailed.

#### Coding and reliability analysis

All participants were videotaped. During the distribution processes (both the puppet’s and the child’s) the experimenter left the room and watched them perform via a Mini-DV-recorder that was placed outside of the study room. If children asked why the experimenter left the room she would simply reply “so that you can do this in privacy”. The children’s behavior was coded live, as Experimenter 1 wrote down how many gummy bears the children had in their plastic dishes after they had completed the action (giving or taking). A randomly selected sample of 20% (15 children from each age group) was coded by a second coder from video. Inter-observer reliability was very high (k = 0.96).

### Results

**Fig 1:**
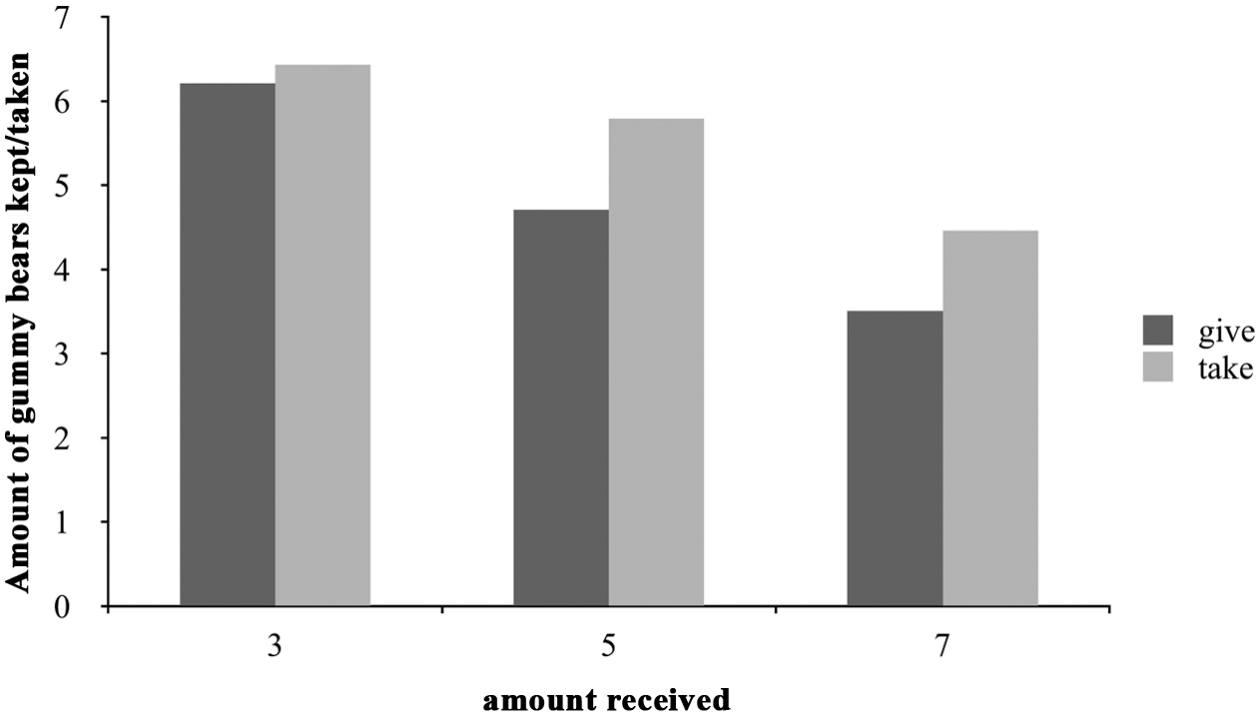
Overview of the three different games. The figure shows the mean amounts of gummy bears in the children’s possession after giving and after taking for three- and five-year-olds combined in all six conditions as defined by the act type (giving: black bars; taking: grey bars) and the amount of gummy bears children had received from the puppet.

To answer our first question about reciprocation in general, we looked at the mean amount of gummy bears children had left after giving to/having taken from the puppet. A three (amount received: 3, 5 or 7 gummy bears) X two (act type: give, take) X two (age: 3 or 5 years) ANOVA yielded a main effect of both factors: amount received, *F*(2, 141) = 35.72, *p* < 0.001, η^2^ = .32, and act type, *F*(1, 142) = 10.98, *p* < 0.001, η^2^ = .049. Thus, the more candies children ended up with after Lola’s act, the more Lola ended up with after their act- so there was reciprocation in terms of amount given. Bonferroni post hoc tests revealed that children who had received three gummy bears had more gummy bears after giving/taking than those who had received five and those who had received five had more than those who had received seven (both *p*s ≤ 0.001, two-tailed). In addition, there was a main effect of act type that suggests that children overall kept fewer gummy bears for themselves—and so shared more with Lola—when Lola had previously *given* gummy bears to them rather than *taken* gummy bears from them (see Fig 1). There was neither a main effect of age nor were there any interactions.

Additionally, we investigated whether the children’s reciprocal behavior differed from how the puppet had treated them. Only significant differences are reported: In the *give 3* condition, five-year-olds kept significantly less than seven gummy bears after giving to the puppet (*M* = 6.3, *t*(11) = -2.39, *p* = 0.036, *d* = 0.980, two-tailed); in the *take 3* condition they took significantly more than three (namely, *M* = 5.1 gummy bears, *t*(11) = 3.44, *p* = 0.006, *d* = -1.404, two-tailed). Hence, in both of these conditions, five-year-olds showed a competing tendency towards equal distributions that three-year-olds did not show.

**Fig 2:**
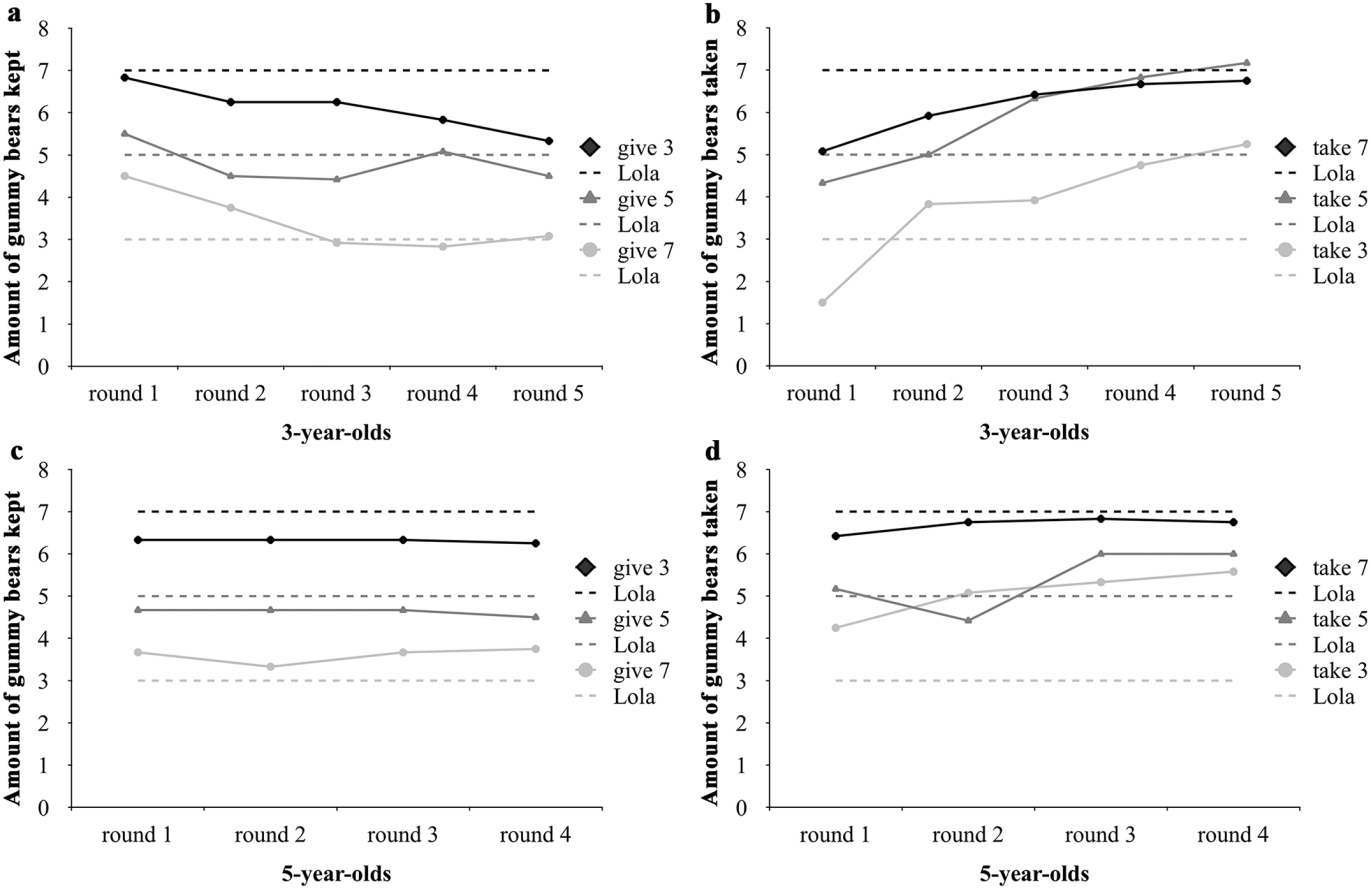
Overview of the reciprocal behavior over the course of the game. In each condition, the reciprocal behavior of the children can be compared to a dotted line of the same color that represents the amount of gummy bears the puppet Lola gave to the children. Sections **a** and **c** refer to the giving conditions of both age groups and hence show the amount of gummy bears kept by the children, sections **b** and **d** refer to the taking conditions, i.e., show the amounts of gummy bears taken by the children. Section **a** shows the development of the giving behavior of the three-year-olds. The figure shows a slight decline in amounts of gummy bears kept for themselves, i.e., a more generous behavior over time, at least in the give 3 and give 7 conditions. In section **b**, it is shown how the amount of gummy bears kept increased over the course of the game Therefore, in all taking conditions, they became more selfish. Sections **c** and **d** show the reciprocal behavior of the five-year-olds. Their reciprocal behavior only changed over time in the taking conditions, where they tended to take more over the course of the game, and most so in the take 5 condition (**d**). In the giving conditions (**c**), their reciprocal behavior stayed rather constant and more closely resembled that of the puppet Lola.

We also examined whether the reciprocal behavior of the children changed over the course of the game. As the three- and five-year-olds differed in the amount of rounds they played (5 and 4 rounds, respectively), we analyzed both age groups separately with a repeated measures ANOVA with round as the within-subjects factor, and act type (giving or taking) and amount received (3, 5 or 7 gummy bears) as between-subject factors. Sphericity was not given for either age group (three-year-olds: Mauchly W = 0.462, χ^2^(9) = 49.710, *p* < 0.001; five-year-olds: Mauchly W = 0.678, χ^2^(5) = 25.187, *p* < 0.001), so Greenhouse-Geisser corrected values are reported. For the three-year-olds, there was a significant effect of round, *F*(2.870, 189.451) = 3.095, *p* = 0.030, η^2^ = 0.045, and an interaction between round and act type, *F*(2.870, 189.451) = 20.495, *p* < 0.001, η^2^ = 0.237. The amount of gummy bears children had left after giving decreased, which means that they gave more over the course of the game. The amounts of gummy bears taken increased as well, which means that children in the taking conditions became more selfish. In this analysis, the only significant between-subject factor was amount received, *F*(2, 66) = 17.155, *p* < 0.001, η^2^ = 0.342 (see above). For the five-year-olds, there was a significant effect of round, *F*(2.386, 157.459) = 5.036, *p* = 0.005, η^2^ = 0.071, and also an interaction between round and act type, *F*(2.386, 157.459) = 5.607, *p* = 0.003, η^2^ = 0.078; the amounts given overall stayed rather constant, the amount taken increased. In this age group, both between-subject factors were significant (amount received: *F*(2, 66) = 20.980, *p* < 0.001, η^2^ = 0.389; act type: *F*(1, 66) = 11.869, *p* = 0.001, η^2^ = 0.152; see above). Fig 2 gives an overview of the changes in sharing behavior for both age groups.

### Discussion

The current study produced two new findings. First, children were affected by the number of gummy bears they ended up with after the partner acted: The more they ended up with, the more generously they reciprocated. Previous studies have shown a preference for others who reciprocate (e.g., [18]) and a general tendency toward reciprocation [7], but this is the first evidence that children’s reciprocal behavior is affected by the amount of resources previously delivered to them. Second, children’s reciprocal behavior was affected by the type of act: Children were more generous when they were left with a certain amount after an act of *giving* than when they were left with that same amount after an act of *taking*. This presumably reflects some judgment of the partner’s social intentions, or, alternatively the social framing of the act as a friendly or unfriendly one. These findings are consistent with of the results reported by [14] with adults. As in that case, the three-year-olds also became more generous in the giving conditions and more selfish in the taking conditions, while the five-year-olds only became more selfish in the taking conditions. This further supports their framing of the puppet’s actions as friendly vs. unfriendly. A similar pattern of behavior was also found by [14] in adults: Dictators in the taking condition did not take much in the beginning of the game, but took more as the game continued, while donations of dictators in the giving condition remained stable (Experiment 4). However, the question remains why the three-year-olds show both effects (becoming more generous in the giving conditions and becoming more selfish in the taking conditions), and the five-year-olds only seemed to be affected in the taking conditions. We could speculate that this could have to do with the competing tendency of the five-year-olds to make equal splits that we were able to identify in our study: In two of the unequal conditions (i.e., give 3 and take 3), the five-year-old children’s reciprocal behavior showed a tendency towards equality. This is consistent with previous findings (e.g., [19]). It thus seems likely that a competing tendency for the older children in our study—somewhat weaker than the factors we manipulated and measured, i.e., experiencing being given vs. having various amounts of resources taken away—was a tendency toward equality. In completely neutral contexts with no previous history, five-year-old children prefer equal splits of resources (e.g., [12; 20]), and this factor thus helps to provide a fuller explanation of all of our results across the conditions.

Given that even young infants are surprised by resource distributions that are not numerically equal (e.g., [20; 21]), one could imagine that young children’s reciprocity is based on some non-social judgment about the number of resources distributed. The current results show that this is clearly not the case. Studies focusing on other aspects of children’s behavior have found that their assessments of other people’s intentions are of critical importance. For example, both [22] and [23; 24] found that young children are less likely to behave prosocially toward an actor who had previously done something, or even intended something, antisocial. But in the current study, it was not the case that the partner had acted antisocially—indeed, in all conditions the puppet shared resources with the child—but rather that her sharing behavior resulted from an act typically viewed as manifesting prosocial intentions (giving) or antisocial intentions (taking).

The most general implication is that children’s judgments about resource distributions, and their reciprocation, are not only based on numerical calculations of resources, but rather on the social implications of the distributive act itself. However, we cannot rule out that the current results are not merely due to having framed the actions as personal gains and losses. To rule out this possibility, we conducted a follow-up study in which gummy bears were obtained by winning or losing a lottery.

## Study 2

To further ensure that children made their choices in Study 1 based on their experience of having goods taken from them or given to them, we conducted a second study in which children played a game where they won or lost gummy bears from/to a puppet. Following the idea of [14] (Experiment 5), the goal of this study was to explore children’s behavior when similar distributions occurred that could potentially be framed as personal gains or losses but without any differing social intentions on the part of the partner.

### Methods

#### Participants

Children whose parents had previously given written consent were recruited from and tested in various kindergartens in Kassel, Germany and surrounding towns. Unfortunately, parents did not give consent to videotape their children. Twenty-two children of three years of age (ten boys, twelve girls) and 24 children of five years of age (eleven boys, 13 girls) took part in this study. The three-year-olds age ranged from 37 to 47 months with a mean age of 42.09 months (SD 2.91 months). The five-year-olds age ranged from 59 to 71 months with a mean age of 64 months (SD 3.05 months). The children were from broadly middle-class backgrounds.

#### Study setup and design

Study materials were similar to Study 1 and consisted of a hand puppet (45 cm tall), a blue and a beige placemat, two small plastic dishes, two opaque plastic boxes, a memory game, and gummy bear candies. Additionally, a plastic bowl was used to draw numbers from. The study setup was very similar to Study 1. Each child was introduced to the experimenter and a puppet named Lola (played by the second experimenter) in her classroom and then went to the study room with them. In the study room, the child, Lola and the experimenter played a memory-like game for a warm up. After that, the experimenter asked the child to sit down at the table in front of the blue felt placemat and Lola to sit down in front of the beige one, facing each other at the table, and showed them the plastic dishes and boxes. Depending on the condition, either the puppet or the child was given ten gummy bears. Then a number was drawn from a plastic bowl, determining how many gummy bears the child would receive from the puppet’s resources (winning condition) or how many the child would lose to the puppet (losing condition). After five complete rounds, the experimenter asked Lola and the child to show them how many gummy bears they had and exchanged those for new ones.

In the winning condition, each play round started out with the puppet Lola receiving ten gummy bears from the experimenter. The experimenter then announced that she would now draw a number from her bowl, which would determine how many gummy bears the child gets from Lola’s gummy bears. Each time, she drew the number five, therefore, in each round, the child won half of the puppet’s candies. The experimenter then transferred five of Lola’s candies to the child and asked both players to count the gummy bears and then store them in their boxes. Then, the child received ten new gummy bears from the experimenter, who told the child that this time, she would not draw a number but the child could give as many gummy bears to Lola as she liked. During the child’s actions, the experimenter turned her back and took notes. After the child was done, the gummy bears were again counted and put away.

In the losing condition, each play round started out with the child receiving ten gummy bears from the experimenter. The experimenter then announced that she would now draw a number from her bowl, which would determine how many gummy bears the puppet would get from the child’s ten. Each time, she drew the number five, therefore, in each round, the child lost half of her gummy bears to the puppet Lola. The experimenter then transferred five of the child’s candies to Lola and asked both players to count the gummy bears and then store them in their boxes. Now the puppet received ten gummy bears from the experimenter. The experimenter told the child that this time, she would not draw a number but the child could decide how many gummy bears she wanted to take from Lola. After the child was done, the gummy bears were again counted and put away.

#### Coding

As we did not have permission to videotape children, their actions were coded live by Experimenter 1. The experimenter wrote down how many gummy bears the children had in their plastic dishes after they had completed the action (giving or taking).

### Results

**Fig 3:**
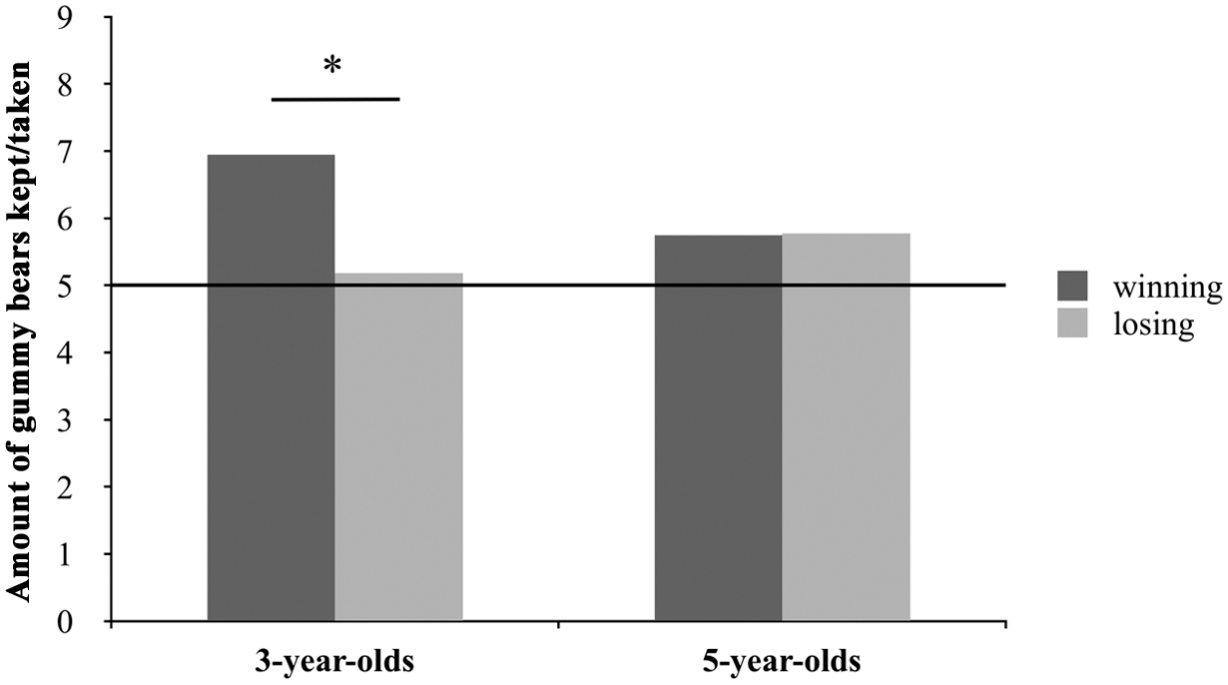
Overview of the results of Study 2. Three-year-olds had significantly more gummy bears left after giving to the puppet in the winning condition than what they had received, hence, they gave the puppet less than five gummy bears after winning five from her.

To compare the reactions to winning and losing we performed a two (condition: winning vs. losing) X two (age: 3 or 5 years of age) ANOVA. Neither condition nor age significantly influenced the children’s reciprocal behavior. Children of both age groups did not have more than five gummy bears left on average, except for the three-year-olds in the winning condition: By having seven gummy bears left on average, they gave the puppet significantly *less* than five gummy after they had won gummy bears from her, *t*(11) = 2.54, *p* = 0.027, *d* = 1.038, two-tailed (see Fig 3).

**Fig 4:**
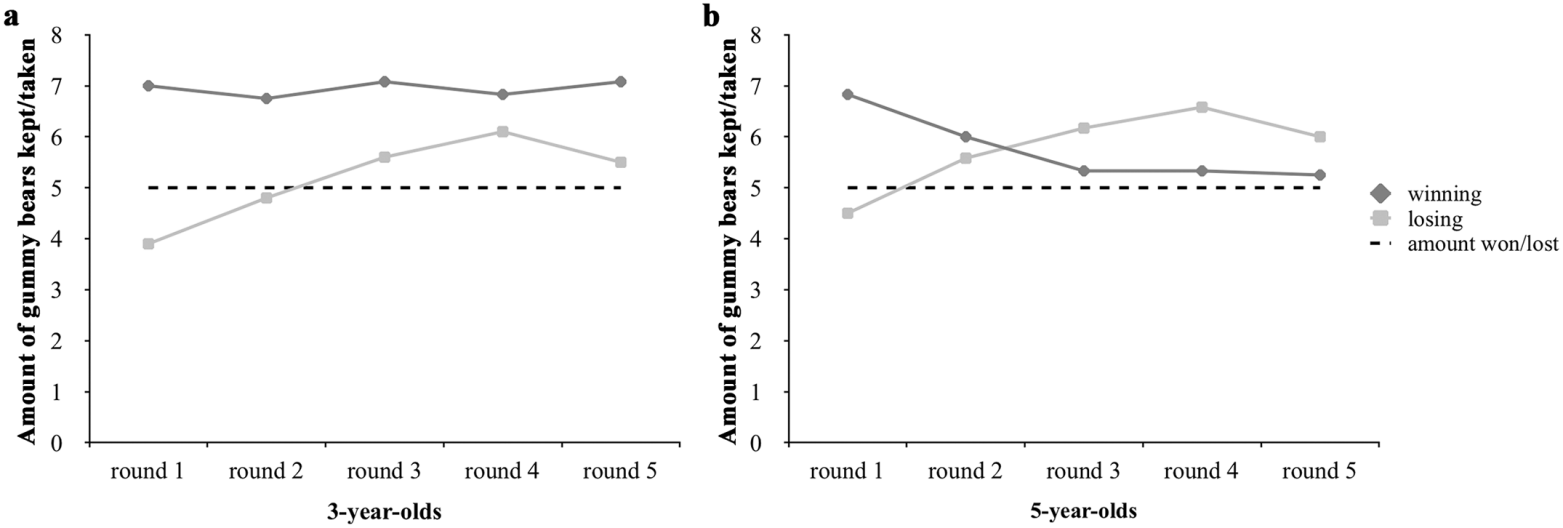
Overview of the reciprocal behavior over the five rounds. Section **a** shows the three-year-olds reciprocal behavior over the course of the game in comparison to the amount they had won/lost (dotted line). While the descriptive data suggests that the three-year-olds kept more for themselves in the losing condition, this change is not significant. As section **b** shows, the reciprocal behavior of the five-year-olds changed depending on the condition. Over the course of the game, five-year-olds in the winning condition tended to have less gummy bears left, hence, gave more, and the five-year-olds in the losing condition tended to take more.

Additionally, we also examined whether the reciprocal behavior of the children changed over time. We performed repeated measures ANOVAs with round as the repeated factor and condition as the between-subject factor separately for both age groups to match the analyses from Study 1. As sphericity was not given (three-year olds: Mauchly W = 0.253, χ^2^(9) = 25.334, *p* = 0.003; five-year-olds: Mauchly W = 0.179, χ^2^(9) = 35.122, *p* < 0.001), all values reported are Greenhouse-Geisser corrected. There were no effects of round or condition and no interactions between the factors for the three-year-olds. For the five-year-olds, there was a significant interaction between round and condition, *F*(2.147, 47.232) = 9.424, *p* < 0.001, η^2^ = 0.300, but no main effects. Fig 4 shows the sharing behavior over the five rounds.

### Discussion

Children did not show different reactions to winning and losing resources. This further suggests that the puppet was not perceived as being responsible for the outcomes in this follow-up study and thus the children did not ascribe social intentions to her. These findings are consistent with those of [14] for adults who were also not affected by winning vs. losing—adults did also not reciprocate differently after winning money vs. losing money. Additionally, the younger participants in our study reciprocated significantly less gummy bears to the puppet than they had previously won, further suggesting that they did not view the puppet as being responsible for the amount of candies the children obtained in each round. The behavior of the five-year-olds changed over time as a result of the condition that they were placed in—in the winning condition, they became more generous over time, in the taking condition, they became more selfish, although there were no main effects of round or condition. However, we cannot completely determine whether the children viewed Lola as not responsible for their outcomes because of the lottery draw or because the second experimenter carried out the giving vs. taking action for her.

## General Discussion

In general, human beings, including children, are motivated to obtain resources. The problem is that others around them have the same motivation. Given this situation, reciprocity is a way for social organism to obtain more resources over time (e.g., [25]). That is, one chooses to interact with and to share with those who are likely to do the same in return, and this is beneficial for both partners in the long run. In order to reciprocate with the right people, i.e., those who have not provided help or resource against their will or by accident, but instead have shared and helped intentionally, humans must have developed various methods for assessing the social intentions of others. Our question here was if these methods for assessing social intentions are already present and exercised by preschool children.

Our studies provide an affirmative answer to this question. Three- and five-year-old children indeed do not just blindly reciprocate based on some numerical calculation to all social partners. They reciprocate selectively toward those who have shared with them based on cooperative intentions. [13] has pointed out that if the main motivation behind wanting a “fair share” were simply to get more resources, then we could not explain why people are not just unhappy at receiving less than a fair share but positively resentful. They are happy to receive X resources in general, but if others get more they feel they have been treated without due respect. In the current study, the children seemingly felt like the puppet was either treating them cooperatively or uncooperatively, and they did not want to continue interacting in the long run with an uncooperative partner (so they reciprocated less generously). Importantly, in our follow-up study (Study 2) we effectively ruled out an explanation in terms of the child seeing the resources she obtained as either personal losses or personal gains. Children perceived the situation as a social interaction between partners and responded accordingly.

The current studies thus contributes to a growing literature that suggests that while preschool-aged children are not very articulate in talking about moral issues and/or making explicit moral judgments, they are already to some degree moral agents (see [26], for a review). Based on the current results, in combination with other recent results on social phenomena such as procedural justice, we may conclude that children’s reactions to the distribution of resources is not so much about the amounts of resources shared, and their desire to obtain more of them, but rather about how they are being treated as a social partner.

## Supporting Information

S1 Dataset Dataset of Study 1. (XLSX)

S2 Dataset Dataset of Study 2. (XLSX)
